# Noninvasive Hemodynamic Assessment with Impedance Cardiography During Spinal and Epidural Anesthesia in Obstetrics

**DOI:** 10.3390/jcm15010074

**Published:** 2025-12-22

**Authors:** Łukasz Czyżewski, Małgorzata Juda, Justyna Teliga-Czajkowska, Janusz Wyzgał, Janusz Sierdziński, Andrzej Silczuk, Łukasz Dudziński

**Affiliations:** 1 Department of Geriatric Nursing, Faculty of Health Sciences, Medical University of Warsaw, 02-007 Warsaw, Poland; lukasz.czyzewski@wum.edu.pl; 2 Institute of Nursing, Faculty of Health Sciences and Psychology, Collegium Medicum, University of Rzeszow, 35-310 Rzeszow, Poland; malgorzata.juda@interia.pl; 3 Department of Obstetrics and Gynecology Didactics, Faculty of Health Sciences, Medical University of Warsaw, 00-575 Warsaw, Poland; justyna.teliga-czajkowska@wum.edu.pl; 4 Department of Nephrology Nursing, Faculty of Health Sciences, Medical University of Warsaw, 02-007 Warsaw, Poland; janusz.wyzgal@wum.edu.pl; 5 Department of Medical Informatics and Telemedicine, Medical University of Warsaw, 00-581 Warsaw, Poland; janusz.sierdzinski@wum.edu.pl; 6 Department of Community Psychiatry, Medical University of Warsaw, 02-353 Warsaw, Poland; 7 Department of Emergency Medical Services, Faculty of Health Sciences, Medical University of Warsaw, 02-091 Warsaw, Poland; lukasz_dudzinski@o2.pl

**Keywords:** impedance cardiography, obstetric anesthesia, spinal anesthesia, epidural analgesia, hemodynamics, vasopressor strategy, phenylephrine, ephedrine, stroke volume, cardiac index

## Abstract

**Background/Objectives**: Spinal anesthesia (SA) for cesarean section and epidural analgesia (EA) for vaginal delivery induce hemodynamic changes that may compromise maternal and fetal safety. In this observational, hypothesis-generating study, we used impedance cardiography (ICG) to characterize maternal hemodynamic responses to EA for labor versus SA for cesarean delivery and to describe hemodynamic profiles associated with commonly used local anesthetic and vasopressor regimens. **Methods**: In this observational study, 132 women at term were included (52 with epidural analgesia (EA), 80 with spinal anesthesia (SA)). Hemodynamic parameters were measured using the ICON electrical cardiometry monitor (Osypka Medical GmbH). ICON and oscillometric blood pressure (BP) monitoring captured cardiac index (CI), stroke volume (SV), heart rate (HR), systemic vascular resistance index (SVRI), and thoracic fluid content (TFC) at T0 (baseline), approximately 5 and approximately 10 min, skin incision, delivery, and oxytocin administration. **Results**: CI remained stable and comparable between EA and SA (3.9 ± 0.6 vs. 3.9 ± 0.6 L/min/m^2^; *p* = 0.530). SV was higher in EA (85.1 ± 11.3 vs. 78.1 ± 9.7 mL; *p* < 0.001), whereas HR was higher in SA (92.2 ± 12.9 vs. 85.8 ± 12.5 bpm; *p* = 0.009). In EA, ropivacaine and bupivacaine showed similar hemodynamic profiles. Within the SA cohort, women managed with phenylephrine infusion had lower CI and HR but higher MAP and SVRI compared with those receiving ephedrine boluses, consistent with the expected pharmacodynamic profiles of these agents. **Conclusions**: ICG was feasible and provided dynamic, noninvasive estimates of maternal cardiovascular adaptation during obstetric anesthesia. In this non-randomized, exploratory cohort, descriptive differences in hemodynamic profiles between vasopressor strategies were more pronounced than between local anesthetics. Phenylephrine-based management showed a pattern of higher BP and SVRI but lower CI and HR, whereas ephedrine-based management tended to preserve CI through chronotropic effects.

## 1. Introduction

Electrical bioimpedance of the thorax (impedance cardiography, ICG) is a noninvasive method of cardiovascular assessment, based on the analysis of changes in tissue electrical resistance in response to a low-intensity, high-frequency current. In recent years, ICG has gained significance as an alternative to invasive methods such as pulmonary artery catheterization, enabling continuous monitoring of hemodynamic parameters including cardiac output (CO), cardiac index (CI), stroke volume (SV), and systemic vascular resistance index (SVRI) [1,2]. Spinal anesthesia (SA), routinely used for cesarean section, leads to vasodilatation, reduced venous return, and arterial hypotension, which may result in insufficient uteroplacental perfusion and potential fetal compromise [3,4]. Similar but usually less pronounced changes may occur during vaginal delivery under epidural analgesia (EA), particularly in the absence of careful fluid management and monitoring of placental perfusion [5]. In these clinical conditions, precise and dynamic cardiovascular monitoring is crucial for preventing complications and optimizing both fluid therapy and vasopressor use [6]. Traditional methods of hemodynamic assessment, such as measurements of blood pressure (BP), including systolic (SBP), diastolic (DBP), and mean arterial pressure (MAP) and heart rate (HR), while useful, do not allow for accurate characterization of dynamic changes in blood flow and cardiac performance. More advanced techniques, including transesophageal echocardiography, thermodilution, or pulse contour analysis, are of limited applicability during labor due to their invasiveness or the requirement for advanced equipment [7,8]. ICG records changes in thoracic tissue electrical resistance induced by the ejection of blood into the aorta. This enables noninvasive, continuous measurement of CI, SV, SVRI and thoracic fluid content (TFC) in real time, with minimal burden for the patient [1,2]. In obstetric anesthesia, SA for cesarean delivery is associated with abrupt sympathetic blockade, vasodilatation, and reduced venous return, which may compromise uteroplacental perfusion, whereas EA for vaginal delivery generally induces milder fluctuations. ICG has been proposed as a noninvasive method for serial, flow-oriented hemodynamic assessment in obstetric anesthesia, but its role in routine clinical decision-making remains uncertain.

In light of these considerations, the primary aim of the present study was to characterize and compare maternal hemodynamic responses to EA for labor and SA for cesarean delivery, using ICG as a noninvasive monitoring tool. The prespecified primary endpoint focused on early post-block CI trajectories. As secondary, exploratory objectives, we described hemodynamic profiles associated with commonly used vasopressor strategies (phenylephrine infusion versus ephedrine boluses in the SA cohort) and local anesthetic regimens (ropivacaine versus bupivacaine in the EA cohort, intrathecal fentanyl versus morphine in the SA cohort). Intrathecal fentanyl and morphine are both standard adjuncts for cesarean delivery but differ in pharmacodynamic profile (rapid-onset, short-acting versus longer-acting, often perceived as hemodynamically more neutral). We therefore included an exploratory comparison of these opioid regimens to determine whether they are associated with discernible differences in CI- and HR-based profiles that might be relevant for women with limited cardiovascular reserve. Rather than formally validating ICG against a reference standard, our intention was to illustrate its feasibility as a pragmatic bedside monitoring tool for serial, flow-oriented monitoring in routine obstetric anesthesia practice.

From a clinical perspective, the choice of vasopressor during SA for cesarean delivery has well-documented implications for maternal CI and neonatal acid–base status, with phenylephrine typically favoring BP stability at the expense of reflex bradycardia and ephedrine tending to preserve CI through combined α/β-adrenergic stimulation. In contrast, labor EA is usually associated with modest systemic circulatory effects, and contemporary practice allows for different local anesthetics with broadly similar analgesic efficacy. At standard obstetric concentrations, ropivacaine and bupivacaine are widely regarded as providing comparable analgesia, but data on whether they differ meaningfully in terms of flow-oriented hemodynamic profiles under continuous CI monitoring are scarce; we therefore prespecified this contrast as an exploratory comparison. Whether specific combinations of vasopressor and local anesthetic meaningfully modify maternal hemodynamic patterns in otherwise healthy parturients remains less clear and was therefore explored in a hypothesis-generating manner in the present study.

To our knowledge, no previous study has used continuous impedance-based CI monitoring to directly compare SA for cesarean delivery with EA for labor within the same pragmatic cohort while simultaneously examining predefined vasopressor and intrathecal opioid subgroups.

## 2. Materials and Methods

### 2.1. Study Design and Setting

This prospective observational single-center study was conducted at an obstetric hospital in Rzeszów, Poland, between September 2019 and November 2022. The study included pregnant women between 38 and 42 weeks of gestation, aged 18–40 years. Exclusion criteria were the presence of a pacemaker, severe pregnancy-related complications, or lack of informed consent. The study protocol was approved by the Bioethics Committee at the Medical University of Warsaw (AKBE/294/2019, issued on 16 September 2019). In accordance with the committee’s decision, informed consent procedures were adapted to the noninvasive and anonymized observational design of the study. All data were collected in anonymized form, without recording personal identifiers; only clinical, demographic, and hemodynamic parameters were analyzed.

### 2.2. Study Population and Anesthesia Protocols

Final analysis included 52 parturients receiving EA for vaginal delivery and 80 parturients undergoing SA for cesarean section. For the primary comparison, patients were categorized into two main clinical groups according to the neuraxial technique: EA for labor and SA for cesarean delivery. Within each main group, we predefined exploratory subgroups based on routinely used drug regimens. In the EA cohort, women were classified according to the local anesthetic regimen (ropivacaine vs. bupivacaine). In the SA cohort, exploratory analyses contrasted (1) intrathecal fentanyl versus intrathecal morphine as opioid adjuncts to hyperbaric bupivacaine and (2) vasopressor strategies (continuous phenylephrine infusion vs. intermittent ephedrine boluses). All subgroup analyses were prespecified as hypothesis-generating and were not powered for definitive between-group comparisons. For EA, all women received an epidural test dose of 3 mL bupivacaine with epinephrine 0.5% (Marcain with adrenaline). The subsequent epidural dosing strategy followed institutional practice and differed by local anesthetic regimen. In the ropivacaine-based group, the initial bolus consisted of 8 mL ropivacaine 2 mg/mL (total dose 16 mg) combined with 1 mL fentanyl solution (concentration per institutional protocol). In the bupivacaine-based group, the initial bolus consisted of 3 mL bupivacaine with epinephrine 0.5% (Marcain with adrenaline; total dose 15 mg) plus 2 mL fentanyl solution and 5 mL 0.9% sodium chloride. Epidural boluses were administered per institutional protocol to maintain adequate labor analgesia. No epidural morphine was used in this cohort. SA for cesarean delivery consisted of intrathecal hyperbaric bupivacaine 0.5% (Marcaine Spinal Heavy; 3.2 mL, total dose 16 mg) combined with an intrathecal opioid additive—either fentanyl 0.2 mL or preservative-free morphine—with the exact opioid dose determined according to institutional protocol. Thus, by design, anesthetic regimens differed between the EA and SA cohorts, as they reflected standard clinical practice for labour analgesia and cesarean anesthesia rather than harmonized experimental protocols.

### 2.3. Perioperative Management

Fluid co-loading consisted of a standardized 500 mL bolus of a balanced crystalloid solution (Optilyte) administered at the time of neuraxial block placement. In cesarean sections, uterotonic management consisted of oxytocin 5 IU i.v. bolus, followed by an infusion of 20 IU over 4 h immediately after fetal delivery. In the SA cohort, vasopressor support consisted of either a continuous phenylephrine infusion (25–50 μg/min, titrated to maintain SBP within ±10% of baseline) or intermittent i.v. ephedrine boluses (5–10 mg) administered in response to SBP < 100 mmHg or a ≥20% decrease from baseline, particularly when accompanied by bradycardia. In the SA cohort, vasopressor management followed routine clinical practice, and these phenylephrine- and ephedrine-based regimens were alternatives rather than combined within the same patient. Vasopressors were administered at the discretion of the attending anesthesiologist to prevent or treat decreases in BP and were not limited to women who had already met the predefined hypotension criterion. For the purposes of the present analysis, SA patients were classified according to the predominant vasopressor strategy (phenylephrine vs. ephedrine) used throughout the cesarean delivery.

### 2.4. Hemodynamic Monitoring

Hemodynamic measurements were obtained using the ICON electrical cardiometry monitor (Osypka Medical GmbH, Berlin, Germany) [9]. Four single-use surface electrodes were placed on the left side of the body-two in the cervical region above the clavicle and two on the thoracic wall at the level of the xiphoid, in accordance with the manufacturer’s instructions. All measurements were performed with the patient in the supine position. The pre-block recording obtained immediately prior to neuraxial anesthesia served as T0 (baseline). The ICON signal includes a proprietary Signal Quality Index (SQI). In accordance with a pre-specified protocol, artefactual segments and epochs with SQI < 85% were excluded from analysis. Such time points were treated as missing and were not imputed. If the SQI dropped below this threshold during acquisition, the recording was temporarily paused for troubleshooting (e.g., electrode contact, patient motion) and resumed once SQI recovered. All analyses were based on per-patient mean values computed exclusively from high-quality segments (SQI ≥ 85%). The following electrical cardiometry–derived indices were recorded and analyzed: CI (L·min^−1^·m^−2^), SV (mL), SVRI (dyn·s·cm^−5^·m^−2^), and TFC (1/kΩ). Non-invasive blood pressure (SBP, DBP, MAP) was measured oscillometrically at 5 min intervals per routine clinical practice; HR and SpO_2_ were monitored continuously.

Recordings were aligned to predefined, clinically relevant peri-procedural time points. T0 (baseline) was defined as a 60 s average obtained immediately before neuraxial block placement, with the patient in the supine position and before fluid co-loading or vasopressor administration. T1 and T2 were defined as 60 s averages centered on 5 ± 1 min and 10 ± 1 min after completion of neuraxial block placement, respectively. In the SA cohort, skin incision was defined as the moment of the first surgical incision, and the corresponding ICON values were averaged over a 60 s window centered on that event. Delivery was defined as fetal extraction, and oxytocin was defined as the 60 s mean centered on the intravenous oxytocin bolus administration.

Non-invasive oscillometric blood pressure monitoring is intermittent by design, whereas electrical cardiometry provides a continuous beat-to-beat signal. To maintain clinical interpretability and to reflect routine practice, we therefore linked each 60 s ICON average to the nearest available oscillometric BP measurement within the same clinical phase (for example, around T1 or T2). In the vast majority of cases the temporal distance between the ICON window and the corresponding BP reading was ≤2 min. No temporal interpolation of BP values was performed. This approach mirrors standard perioperative hemodynamic assessment, in which continuous CI surrogates are interpreted alongside intermittent non-invasive BP measurements.

Importantly, each predefined time point represents the net hemodynamic state during a clinically meaningful phase rather than an artifact-free, intervention-free moment. Around T0–T2 and skin incision, changes in preload and afterload may reflect both the direct effects of the neuraxial block and concomitant interventions (fluid co-loading, vasopressors) as well as endogenous responses to surgical stimulation. Around delivery and oxytocin administration, uterine contraction, autotransfusion, blood loss, and oxytocin-induced vasodilation all contribute to the observed hemodynamic profile. Our intention was not to isolate the pure pharmacodynamic effect of a single drug at an exact clock time, but to characterize the overall pattern of maternal hemodynamics across these routinely encountered peri-procedural phases.

Hemodynamic data were exported from the ICON device and the anesthesia record, merged by patient and time stamp, and pre-specified inclusion rules (SQI ≥ 85%, removal of artifacts, per-patient averaging) were applied prior to statistical analysis. All unit conventions were kept constant across tables and figures to ensure internal consistency and reproducibility.

### 2.5. Study Endpoints and Hypotheses

This study was designed to compare maternal hemodynamic responses to EA for labor and SA for cesarean delivery using ICG. The primary endpoint was the between-group difference in the early post-block trajectory of CI from baseline to 10 min after neuraxial block, defined a priori as the change from baseline at 5 min (ΔCI_5_ = T1 − T0) and 10 min (ΔCI_10_ = T2 − T0), as well as the mean early change ((ΔCI_5_ + ΔCI_10_)/2). Given the pragmatic, single-center nature of the study and the absence of a formal sample-size calculation, all analyses were considered exploratory and hypothesis-generating.

These trajectories were analyzed using a mixed-design repeated-measures ANOVA with a between-subjects factor group (EA vs. SA) and a within-subject factor time, including their interaction (group × time). Key secondary endpoints comprised analogous trajectories (Δ from T0 and absolute levels at T0, T1, T2) for SV, HR, MAP, and SVRI when comparing EA vs. SA, together with prespecified within-cohort contrasts: in SA, (1) phenylephrine infusion versus ephedrine boluses and (2) intrathecal fentanyl versus intrathecal morphine (with hyperbaric bupivacaine); in EA, ropivacaine versus bupivacaine. TFC was assessed as a supportive index of volume status. Hypotension was defined primarily as a ≥20% decrease in SBP from the individual baseline at any time after block (with an exploratory threshold of SBP <100 mmHg), and was analyzed both as unadjusted incidence and via multivariable logistic regression reporting odds ratios (OR) with 95% CIs; prespecified covariates for risk adjustment were baseline MAP, baseline CI and baseline HR. A priori hypotheses specified that EA and SA would differ in early CI trajectory; within SA, phenylephrine would be associated with fewer hypotensive episodes, lower HR, and higher MAP/SVRI versus ephedrine; within SA, intrathecal fentanyl (vs. morphine) would yield higher CI/HR with similar MAP/SVRI; and within EA, ropivacaine and bupivacaine would show broadly comparable hemodynamic profiles with a priori expectation of slightly higher SV with ropivacaine. Secondary and exploratory endpoints were interpreted descriptively (exact *p*-values and 95% CIs reported without formal multiplicity adjustment). Accordingly, emphasis was placed on the direction and magnitude of effects rather than on dichotomous significance testing.

### 2.6. Statistical Analysis

Normality was assessed using the Shapiro–Wilk test, and homogeneity of variance with Levene’s test. For between-group comparisons, independent samples *t*-tests were applied with Welch’s correction where appropriate. Repeated-measures ANOVA was used to evaluate within- and between-subject effects across serial time points (defined as baseline before anesthesia [T0], 5 min after anesthesia [T1], 10 min after anesthesia [T2], and subsequent clinically relevant perioperative milestones), considering anesthesia type, time, and their interaction as factors. When sphericity was violated, Greenhouse–Geisser correction was applied. A two-sided *p*-value of less than 0.05 was considered statistically significant. For binary outcomes (e.g., hypotension ≥ 20% SBP drop), unadjusted odds ratios with 95% CI were calculated. In addition, multivariable logistic regression models were fitted as specified above. No formal a priori sample size calculation was performed; the study sample reflects the number of eligible parturients recruited during the study period and thus represents a convenience sample. Consequently, the trial was not powered to detect small between-group differences, and all inferential analyses should be interpreted as exploratory. Subgroup sample sizes for the different local anesthetic, intrathecal opioid, and vasopressor regimens (e.g., 40 vs. 40 in the SA subgroups and 26 vs. 26 in the EA subgroups) were likewise determined by consecutive clinical practice patterns rather than by a priori power calculations, and are therefore best interpreted as pragmatic, exploratory contrasts. Primary between-group comparisons of EA versus SA are presented as unadjusted descriptive contrasts. In addition, baseline hemodynamic parameters (CI, SV, HR, MAP, SVRI, TFC) were examined in analysis of covariance models with anesthesia technique as the main factor and maternal age and gestational age as covariates; adjusted differences are reported in the Results. These models were intended to assess the potential impact of demographic differences rather than to provide definitive causal estimates. Effect estimates are reported with 95% CI; multiplicity in secondary/exploratory endpoints was handled descriptively. Given the number of subgroup comparisons, *p*-values were not adjusted for multiple testing and are therefore best viewed as descriptive rather than confirmatory. Missing hemodynamic values arose primarily from transient loss of ICG signal quality or occasional absent non-invasive BP recordings at individual time points. For continuous outcomes, repeated-measures analyses used all available observations under a missing-at-random assumption, without interpolation or imputation of missing values. For binary outcomes such as hypotension, analyses were restricted to patients with complete SBP data; missing BP values were not imputed. Statistical analysis was performed using Statistica 13.3 (Tibco Inc., Tulsa, OK, USA).

## 3. Results

### 3.1. Patient Characteristics

Groups differed in maternal age (EA 28.7 ± 4.4 vs. SA 31.3 ± 4.3 years; *p* = 0.001) and gestational age (EA 39.7 ± 1.0 vs. SA 38.7 ± 0.7 weeks; *p* < 0.001). Body weight was comparable (EA 76.9 ± 13.1 vs. SA 77.3 ± 13.6 kg; *p* = 0.838) (Table 1). In the EA group, local anesthetics were evenly split between ropivacaine and bupivacaine (26/52 each); an epidural opioid additive was documented as fentanyl in 26/52 (50%), while no epidural opioid was documented in 26/52 (50%); epidural morphine was not used. In the SA group, anesthesia consisted of hyperbaric bupivacaine 0.5% with either intrathecal fentanyl (40/80, 50%) or intrathecal morphine (40/80, 50%). Vasopressor management in SA included continuous phenylephrine infusion or ephedrine boluses (each 40/80, 50%). Accordingly, EA–SA comparisons in this study should be interpreted as descriptive contrasts of overall hemodynamic patterns associated with these routine clinical protocols rather than as head-to-head tests of identical anesthetic regimens.

SV was significantly higher in the EA group compared with SA (85.0 ± 11.1 vs. 78.3 ± 9.8 ml; *p* < 0.001)—Table 2.

### 3.2. Spinal Anesthesia (SA)—Anesthetic Agent

We compared women receiving hyperbaric 0.5% bupivacaine with either intrathecal fentanyl or intrathecal morphine. CI and HR were numerically higher in the fentanyl group (4.0 ± 0.7 vs. 3.7 ± 0.6 L/min/m^2^; 94.6 ± 12.8 vs. 88.9 ± 13.5 bpm), but these differences were not statistically significant (*p* = 0.063 and *p* = 0.055, respectively). No significant differences were observed in SV, MAP, SVRI, or TFC (*p* ≥ 0.087; Table 3).

### 3.3. Epidural Analgesia (EA)—Anesthetic Agent

We compared women receiving ropivacaine versus bupivacaine. No statistically significant differences were observed in CI, SV, HR, MAP, SVRI or TFC between these groups (all *p* ≥ 0.256), and the detailed numerical data are therefore not shown to avoid redundancy.

### 3.4. Spinal Anesthesia (SA)—Vasopressor Strategy

A comparative analysis of patients managed with phenylephrine versus ephedrine as the primary strategy for arterial pressure support revealed clear differences in compensatory hemodynamic mechanisms. In the phenylephrine group, CI was significantly lower (3.7 ± 0.6 vs. 4.0 ± 0.6 L/min/m^2^; *p* = 0.017), as was HR (89.2 ± 11.9 vs. 95.3 ± 13.4 bpm; *p* = 0.032). At the same time, MAP was higher (95.4 ± 8.4 vs. 90.8 ± 9.3 mmHg; *p* = 0.024), as was SVRI (2153.4 ± 364.9 vs. 1886.3 ± 399.8 dyn·s·cm^−5^/m^2^; *p* = 0.003). TFC remained stable and did not differ between groups. These findings point to distinct hemodynamic profiles of the two agents: phenylephrine maintains blood pressure primarily through an increase in SVRI and reflex bradycardia, whereas ephedrine supports CI via adrenergic stimulation and tachycardia—Table 4.

### 3.5. Hypotension and Risk Factors

Hypotension, defined as a ≥20% decrease in SBP from baseline, occurred in 17 of 52 patients receiving EA (32.7%) and in 44 of 78 patients receiving SA (56.4%) among women with complete SBP data (the overall SA cohort comprised 80 patients, but 2 had incomplete SBP recordings for this analysis). Within the SA cohort, phenylephrine infusion was associated with markedly fewer hypotensive episodes compared with ephedrine boluses (11/38 [28.9%] vs. 33/40 [82.5%], complete SBP data). When hypotension was alternatively defined as SBP < 100 mmHg, the criterion identified nearly all patients as hypotensive and therefore lacked discriminatory value; subsequent analyses focused on the relative decrease from baseline (≥20%). Because the onset of hypotension and the initiation of vasopressor therapy could occur at varying times after block placement, these events were not time-locked to specific ICON epochs and were analyzed separately from the serial CI/SV/HR trajectories.

In multivariable logistic regression adjusting for baseline MAP, CI, and HR, SA remained independently associated with a higher risk of hypotension (OR 3.48; 95% CI 1.55–7.83). Higher baseline MAP also predicted hypotension (OR 1.05 per mmHg; 95% CI 1.00–1.09), while baseline CI and HR were not significant predictors—Table 5.

### 3.6. Hemodynamic Parameters

#### 3.6.1. Cardiac Index

**Figure 1:**
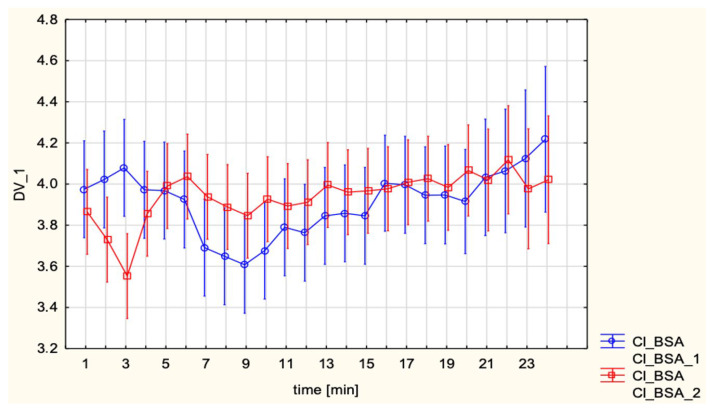
Cardiac Index (CI, L/min/m^2^) in patients under spinal anesthesia (cesarean section) and epidural analgesia (vaginal delivery).

In repeated-measures analysis, CI did not differ significantly between SA and EA (group effect *p* = 0.443, time *p* = 0.148, interaction *p* = 0.168). In keeping with these findings, auxiliary *t*-tests yielded *p*-values above the pre-specified 0.05 threshold (e.g., *p* = 0.055 for selected time-point contrasts). Overall, CI values remained stable throughout the observation period (Figure 1).

#### 3.6.2. Stroke Volume

**Figure 2:**
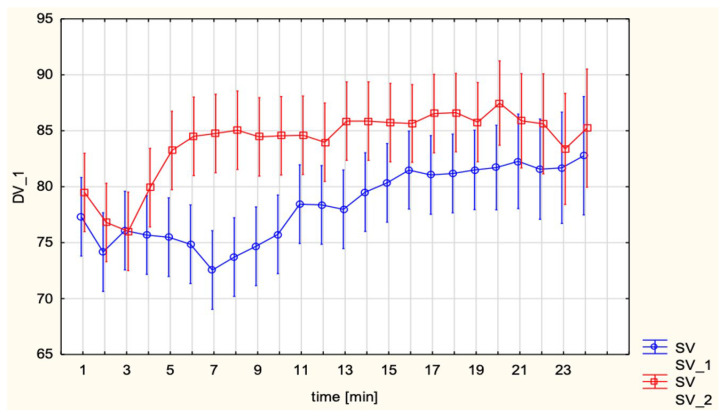
Stroke Volume (SV, mL) under spinal versus epidural anesthesia.

SV was higher in EA than SA (85.1 ± 11.3 vs. 78.1 ± 9.7 mL; *p* = 0.0004). Repeated-measures analysis revealed no significant time effect (*p* = ns) or group × time interaction (*p* = ns) (Figure 2).

#### 3.6.3. Heart Rate

**Figure 3:**
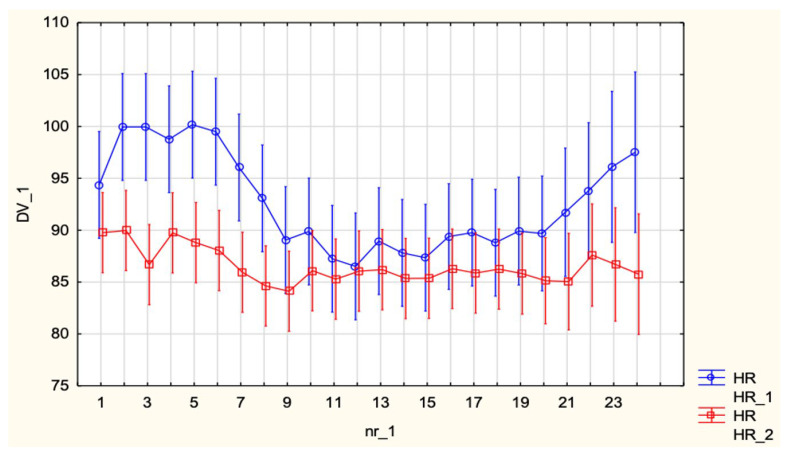
Heart Rate (HR, bpm) in patients undergoing spinal anesthesia for cesarean section and epidural analgesia for vaginal delivery.

HR was significantly higher in SA compared with EA (92.2 ± 12.9 vs. 85.8 ± 12.5 bpm; *p* = 0.0047). No group × time interaction was observed (Figure 3).

#### 3.6.4. Mean Arterial Pressure

**Figure 4:**
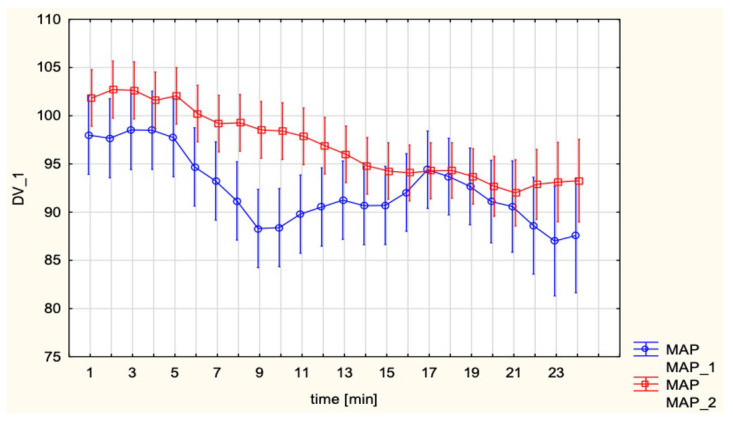
Mean Arterial Pressure (MAP, mmHg) in patients undergoing spinal anesthesia for cesarean section and epidural analgesia for vaginal delivery.

MAP did not differ significantly between groups (EA 95.7 ± 8.6 vs. SA 92.8 ± 9.3; *p* = 0.070). No significant time effect or interaction was detected (Figure 4).

#### 3.6.5. Systemic Vascular Resistance Index

**Figure 5:**
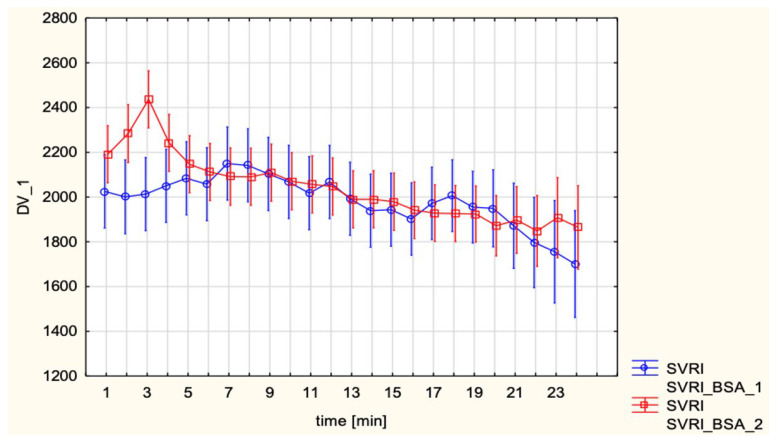
Systemic Vascular Resistance Index (SVRI, dyn·s·cm^−5^/m^2^) after spinal and epidural anesthesia.

SVRI showed no significant difference between groups (2016.4 ± 403.7 vs. 1992.0 ± 351.9 dyn·s·cm^−5^/m^2^; *p* = 0.714). Both time effect and interaction remained non-significant (Figure 5).

#### 3.6.6. Thoracic Fluid Content

TFC remained stable over time and did not differ materially between EA and SA (Table 2).

In summary, CI remained stable and comparable between EA and SA, with higher SV in EA and higher HR in SA, while MAP, SVRI, and TFC showed no relevant differences over the observation period.

## 4. Discussion

The present study evaluated maternal hemodynamics during SA for cesarean section and EA for labor using ICG, a non-invasive method that enables continuous assessment of cardiac performance. Our work builds on earlier experience with impedance-based CI monitoring in obstetric anesthesia, most notably the whole-body ICG study by Tihtonen et al. [10], who continuously recorded CI, HR, MAP and SVRI during elective cesarean section under SA and demonstrated that this technique was technically feasible throughout the procedure. In their cohort of healthy parturients, CI increased by approximately 47% and SVRI decreased by about 39% within 2 min after delivery, while MAP remained relatively stable, illustrating how ICG can capture rapid, delivery-related hemodynamic shifts that are not apparent from intermittent BP measurements alone. In line with these observations, the present study used thoracic electrical cardiometry to obtain beat-to-beat estimates of CI, SV, and SVRI across predefined peri-procedural time points, thereby extending the application of impedance-based monitoring from descriptive single-technique series to a comparative evaluation of SA versus EA and to predefined subgroups defined by vasopressor strategy and intrathecal opioid adjuncts. In this context, our data provide a descriptive comparison of flow-oriented hemodynamic profiles between these commonly used neuraxial techniques and drug regimens. The findings highlight both shared and distinct cardiovascular adaptations between the two anesthetic approaches, with further insights into the influence of intrathecal opioid adjuncts and vasopressor strategies. A key result of this investigation was the stability of CI across both SA and EA groups (3.9 ± 0.6 L/min/m^2^ in each). This stability is consistent with the notion that, in otherwise healthy term parturients managed with standardized fluid and vasopressor protocols, global CI can be preserved despite differences in anesthetic mechanism and degree of sympathetic block. Comparable preservation of maternal CO during cesarean delivery under SA has been demonstrated by Michelsen et al. [11], who used continuous invasive arterial waveform analysis in 71 healthy women and found that median CO before SA (6.51 L/min, IQR 5.56–7.54) was virtually identical to values immediately before delivery (6.40 L/min, IQR 5.83–7.56; *p* = 0.40), despite a modest increase in SBP and a reduction in HR. Collectively, these data suggest that, when SA-induced hypotension is prevented with a standardized low-dose phenylephrine regimen, global maternal CO can be maintained throughout cesarean delivery, in line with the stable CI observed in the SA cohort in the present study. These observations are also in line with broader reviews of maternal hemodynamic monitoring in obstetric anesthesia, which emphasize that neuraxial techniques are usually well tolerated in healthy parturients when accompanied by proactive hemodynamic management. Dyer and James [12] highlighted that, in the setting of SA for cesarean delivery, maintaining maternal blood pressure with vasopressors such as phenylephrine is associated with minimal umbilical arterial base deficit, currently regarded as one of the most sensitive short-term markers of neonatal well-being, and that low-dose spinal regimens combined with prophylactic phenylephrine infusions provide a particularly stable maternal hemodynamic profile.

Recent prospective data using non-invasive CO monitoring with USCOM during elective cesarean section further support this concept: Lambertini et al. [13] showed that locoregional anesthesia led to the expected decrease in BP and HR, whereas CO and CI exhibited only a modest, largely non-significant downward trend, SV recovered after an initial fall, and SVR changed minimally; importantly, 63% of women required vasopressor therapy, yet no cases of neonatal acidosis or neonatal intensive care admission were observed, although lower maternal CI before fetal extraction was associated with umbilical arterial pH < 7.20. Taken together, overall, the available data from these and other series indicate that in healthy term parturients, neuraxial anesthesia is hemodynamically well tolerated when accompanied by structured fluid loading, timely vasopressor administration, and, where available, advanced hemodynamic monitoring, which is consistent with the stable CI observed in both the SA and EA cohorts in the present study.

Interestingly, in unadjusted analyses SV was higher in the EA group compared with SA (85.1 ± 11.3 vs. 78.1 ± 9.7 mL; *p* < 0.001). However, this difference was attenuated and no longer statistically significant after adjustment for maternal age and gestational age (Table 2), and should therefore be interpreted as a descriptive, hypothesis-generating pattern rather than a definitive effect estimate. The direction of this pattern is compatible with the less abrupt sympathetic block associated with EA, which is expected to produce less venous pooling and better preservation of preload. By contrast, the higher HR observed in SA (92.2 ± 12.9 vs. 85.8 ± 12.5 bpm; *p* = 0.009) suggests that tachycardia may have acted as a compensatory mechanism to offset the reduction in SV and thereby maintain CI. A similar interplay between preload, SV, and HR has been described in impedance-based cardiac output studies of SA for cesarean delivery. In twin pregnancies monitored with bioreactance (NICOM), Xu et al. [14] reported that CO decreased by approximately 17.5% at 5 min after SA and remained significantly below baseline until fetal delivery, with SV closely paralleling this decline, whereas HR increased in the early post-spinal period. Total peripheral resistance showed only minimal changes after SA, and the authors concluded that hypotension was mainly driven by reduced venous return and CO rather than by a primary fall in SVR. In the same cohort, phenylephrine boluses increased MAP but further reduced CO by slowing the heart rate, again highlighting the central role of SV and compensatory tachycardia in determining maternal CO under SA. Taken together with Doppler- and invasively derived data in singleton pregnancies, our observations of lower SV and higher HR with preserved CI after SA compared with EA are physiologically plausible and appear consistent with the view that SA tends to induce abrupt preload-related changes with an early increase in HR, whereas epidural techniques by producing a more gradual onset of sympathetic block, are likely to be associated with less abrupt reductions in preload and SV.

These results are consistent with previous hemodynamic observations in obstetric anesthesia, where SA–induced hypotension is now understood to result primarily from a rapid and pronounced decrease in SVR with peripheral vasodilation and venous pooling rather than from a major fall in CO. As summarized by Loubert [15], contemporary Doppler and CO studies in healthy parturients show that, after SA for elective cesarean delivery, peripheral vascular resistance falls markedly while CO is usually preserved or even slightly increased, provided that patients do not receive high-dose phenylephrine infusions and that corrected ejection time remains unchanged, arguing against a large reduction in venous return. This framework is compatible with the present finding that, compared with EA, SA was associated with lower SV and higher HR but a similar CI, suggesting that tachycardia compensated for modest preload-related changes on the background of a sympathectomy-driven fall in SVR. MAP and SVRI did not differ significantly between groups, which can be attributed to the standardized use of vasopressors. However, subgroup analyses revealed distinct differences depending on the choice of vasopressor. Continuous phenylephrine infusion led to lower CI and HR, while maintaining MAP and elevating SVRI, reflecting its pure α-adrenergic vasoconstrictor profile. In contrast, ephedrine, administered in boluses, preserved CI largely through β-adrenergic chronotropic stimulation. This pattern closely matches the pharmacodynamic profiles described by Loubert, who notes that phenylephrine predominantly increases peripheral vascular resistance at the expense of reflex bradycardia and reduced CO, whereas ephedrine increases HR and CO with comparatively smaller effects on SVR.

These hemodynamic patterns also align with randomized trials and reviews comparing phenylephrine and ephedrine that are summarized in the narrative review by Dusitkasem et al. [16]. In healthy parturients undergoing elective cesarean delivery, these studies consistently show that phenylephrine is more effective than ephedrine at maintaining maternal BP and reducing intraoperative nausea and vomiting, and is associated with higher umbilical arterial pH and a lower incidence of fetal acidosis, whereas ephedrine, because of greater placental transfer and β-adrenergic stimulation in the fetus leads to higher neonatal lactate, glucose, and catecholamine levels. In contrast, in high-risk pregnancies with uteroplacental insufficiency or hypertensive disorders, the same review concludes that both phenylephrine and ephedrine appear similarly effective and safe with respect to maternal blood pressure and neonatal acid–base status when used in moderate doses. Against this background, our finding that phenylephrine was associated with higher MAP and SVRI but lower CI and HR, whereas ephedrine preserved CI through chronotropic stimulation, illustrates hemodynamic patterns that are compatible with the expected drug-specific profiles in this largely low-risk cohort. The study was not powered or designed to detect differences in neonatal outcomes, and no adverse neonatal signal was observed.

The present data are compatible with the view that vasopressor choice should be individualized: phenylephrine may be preferable in patients at risk of severe hypotension, whereas ephedrine could be advantageous in women with compromised cardiac reserve where maintaining CI is critical. Given the observational, non-randomized nature of vasopressor allocation in this cohort, our findings should be interpreted as complementary to, rather than a substitute for, existing randomized evidence.

Another novel observation relates to the adjunctive use of intrathecal opioids in SA. In our cohort, the subgroup receiving intrathecal fentanyl exhibited higher CI and HR compared with those given intrathecal morphine, whereas MAP and SVRI were broadly similar. While both drugs are widely used as neuraxial adjuncts for cesarean delivery, their pharmacodynamic profiles differ: fentanyl has a rapid onset and relatively short duration of action, whereas morphine provides longer-lasting analgesia but is often considered to be associated with more stable hemodynamics. However, most of the existing literature on maternal hemodynamics during obstetric SA has focused on the interplay between local anesthetic dose, fluid loading, and vasopressor strategy, with the intrathecal opioid component held constant. In the randomized trial by Langesæter et al. [12], for example, all women received sufentanil as the intrathecal opioid, and detailed invasive monitoring demonstrated that the major determinants of changes in CO and SVR were the bupivacaine dose and the use of prophylactic phenylephrine, rather than the opioid adjunct itself.

To date, very few studies have systematically compared maternal cardiovascular responses between different intrathecal opioids such as fentanyl and morphine in the context of cesarean section. As underscored in a broader editorial overview of maternal hemodynamic monitoring, most clinically relevant obstetric anesthesia research has relied on HR and BP (with or without advanced CO monitoring) to evaluate the effects of fluids, vasopressors, and local anesthetic regimens. Data specifically addressing opioid-related differences in maternal hemodynamics remain sparse and are usually limited to secondary observations within trials primarily designed to assess analgesic quality and side-effect profiles. Our findings therefore suggest that intrathecal opioid selection might influence CI- and HR-based profiles, but the observed differences were modest and did not reach conventional thresholds for statistical significance. In view of the limited sample size, lack of randomization and multiple comparisons, these observations should be regarded purely as hypothesis-generating and cannot support any change in clinical practice.

Future randomized trials incorporating continuous CO monitoring would be valuable to confirm whether these trends represent true pharmacodynamic differences or are partly driven by patient selection and clinical context.

In the EA cohort, hemodynamic profiles between ropivacaine and bupivacaine were largely comparable. This supports prior evidence that both agents provide effective analgesia with minimal differential cardiovascular impact at obstetric doses. Although most contemporary studies of labor EA have not been designed primarily to compare local anesthetics from a hemodynamic standpoint, non-invasive CO monitoring data suggest that the systemic circulatory effects of EA per se are usually modest and strongly modulated by baseline maternal vascular status. In a recent prospective study using USCOM, Giannubilo et al. [17] observed that, in term women receiving epidural levobupivacaine with sufentanil, CO increased slightly after the epidural bolus and rose significantly by the end of the first stage of labor in the overall cohort, while BP remained broadly stable. In this context, current data suggest that, at standard obstetric concentrations, the choice of epidural local anesthetic—whether ropivacaine or bupivacaine—is unlikely to exert a major independent effect on maternal systemic hemodynamics, which are instead more strongly determined by pre-existing cardiovascular status, labor dynamics, and concomitant vasopressor or fluid strategies. Consistent with this, existing comparative studies of ropivacaine and bupivacaine for labor analgesia have largely focused on motor block, sensory profile, and toxicity rather than on detailed cardiovascular responses, and generally report only small or negligible differences in systemic hemodynamics. Against this background, the similar EC-derived profiles observed with ropivacaine and bupivacaine in our EA cohort reinforce the view that, for healthy parturients, the selection between these agents can be guided primarily by analgesic and motor characteristics rather than by concerns about global circulatory impact. Thus, the clinical decision between these agents may hinge more on side effect profiles, for example, degree of motor block, neurotoxicity or duration of action, than on hemodynamic considerations. This is consistent with work on different intrathecal levobupivacaine doses in cesarean delivery, where CI, HR and vascular resistance were broadly similar between regimens, while differences were mainly seen in motor block, vasopressor needs and recovery characteristics [18].

The use of ICG in this study provided a non-invasive, exploratory tool to track maternal hemodynamic patterns over time. Traditional noninvasive monitoring with intermittent blood pressure and HR does not capture the rapid interaction between preload, afterload and cardiac performance during neuraxial anesthesia and delivery. In the randomized trial by D Ambrosio et al. [18] ICG tracked CI, SV and SVRI from before combined spinal EA through the early post-block period, allowing early detection of hypotension and real-time assessment of the response to ephedrine treatment.

Similarly, Tihtonen et al. [10] used whole body ICG during cesarean section and showed that at delivery CI rose by about forty-seven percent and SVRI fell by about thirty-nine percent within two minutes while MAP remained stable, changes that would not be appreciated with blood pressure recordings alone. Taken together, our findings and prior studies suggest that impedance-based techniques, such as ICG, may provide additional, flow-oriented information on maternal cardiovascular adaptation beyond conventional monitoring. Contemporary reviews suggest that minimally invasive CO monitoring may be useful in selected high-risk obstetric patients by allowing clinicians to titrate fluids and vasopressors according to changes in flow rather than pressure alone [12,13]. Our findings add to this literature by illustrating the type of flow-oriented information that ICG can provide during neuraxial anesthesia. At the same time, it should be acknowledged that ICG remains an indirect, model-based technique whose accuracy can be influenced by thoracic geometry, pregnancy-related anatomical changes, and signal quality; careful electrode placement and stringent signal quality criteria, as applied in this study, are essential to maximize reliability. Moreover, in the absence of a reference CI method in our protocol, the present results should be interpreted as demonstrating technical feasibility and pattern description rather than validating ICG for diagnostic accuracy or routine hemodynamic decision-making.

### Limitations

This study has several limitations. First, it was conducted in a single center with a relatively limited sample size, which may affect the generalizability of the findings. Although the number of patients analyzed (52 in the EA group and 80 in the SA group) is larger than in many previous reports, the study was not powered to detect subtle differences in all hemodynamic parameters, and no formal a priori sample size calculation was performed. Second, the use of ICG, while noninvasive and clinically practical, may be less accurate than invasive reference methods. Nevertheless, we minimized measurement error by excluding recordings with a signal quality index below 85%. Third, our analysis was restricted to the immediate peripartum period, without long-term follow-up of maternal or neonatal outcomes. Fourth, the observational, non-randomized design introduces several sources of confounding. SA and EA were applied to inherently different clinical populations (cesarean delivery versus labor), and baseline characteristics such as maternal age and gestational age differed between groups. Similarly, vasopressor strategy and intrathecal opioid choice reflected individual clinician preference rather than random allocation. Although we adjusted for selected baseline hemodynamic variables in the multivariable analysis of hypotension, we cannot exclude residual confounding, and causal inferences regarding the effect of anesthesia technique or drug regimen on hemodynamics are therefore limited. Fifth, multiple subgroup comparisons were performed without formal correction for multiplicity. As prespecified, *p*-values for secondary and exploratory endpoints are presented descriptively and should not be interpreted as confirmatory; the main value of the study lies in generating hypotheses and illustrating hemodynamic patterns rather than establishing definitive treatment effects. Importantly, as an observational study reflecting routine clinical practice, our findings primarily describe real-world variability in anesthetic management and hemodynamic responses rather than controlled effects of predefined protocols. In this context, the results should be interpreted as providing decision-support information that may help clinicians recognize typical hemodynamic patterns associated with different neuraxial techniques, rather than as evidence supporting specific standardized interventions. At the same time, the observed heterogeneity in vasopressor use, anesthetic regimens, and hemodynamic trajectories highlights areas where greater standardization could be explored in future research. Prospective, protocol-driven studies are warranted to determine whether standardized management strategies can reduce variability and improve hemodynamic stability and clinical outcomes. In addition, no invasive or echocardiographic reference method was used to validate ICG-derived CI in this cohort. As a result, our data cannot address the absolute accuracy or agreement of electrical cardiometry, and any implications for clinical decision-making should be considered preliminary. Finally, we did not model time-varying covariates such as individual anesthetic and opioid doses, dynamic fluid balance, or intraoperative blood loss as separate predictors. In clinical practice these factors are highly interrelated and occur in close temporal proximity to neuraxial block, surgical stimulation, uterine contraction and oxytocin administration. As a result, the hemodynamic patterns we report should be interpreted as net effects of these combined influences within each peri-procedural phase rather than as isolated pharmacodynamic responses to a single intervention. These limitations should be considered when interpreting our results.

## 5. Conclusions

Non-invasive electrical cardiometry was feasible and provided continuous, flow-oriented estimates of maternal hemodynamics during both EA for labor and SA for cesarean delivery. CI remained stable in both techniques, but EA was associated with a more favorable SV profile, while SA induced compensatory tachycardia; MAP and SVRI did not differ significantly between techniques. In the SA cohort, the choice of vasopressor determined the hemodynamic profile more than the choice of local anesthetic. Continuous phenylephrine infusion stabilized BP but reduced CI through reflex bradycardia, whereas ephedrine better preserved CI via chronotropic stimulation. These observations, however, arise from a non-randomized, single-center study and should be interpreted as descriptive rather than causal. From a clinical standpoint, our findings are consistent with the view that EA is hemodynamically well tolerated during labor. During cesarean delivery under SA, vasopressor strategy is typically individualized in clinical practice, with phenylephrine often preferred when prevention of hypotension is paramount and ephedrine used when preserving maternal CI is prioritized; the present observational data are compatible with these patterns but cannot establish causality.

ICG may complement standard monitoring by providing additional, noninvasive information on maternal CI and SV, but further studies comparing ICG with invasive or established noninvasive reference methods are needed before its routine use for hemodynamic decision-making can be recommended.

## Figures and Tables

**Table 1:** Demographics and Clinical Characteristics of the study groups.

Parameter	Epidural (*n* = 52)	Spinal (*n* = 80)	*p*
	Analgesia	Anesthesia	
Age (y)	28.7 ± 4.4	31.3 ± 4.3	0.001
Weight (kg)	76.9 ± 13.1	77.4 ± 13.3	0.825
Gestational age (weeks)	39.7 ± 1.0	38.7 ± 0.7	<0.001
Local anesthetic used			
Ropivacaine (epidural)	26/52 (50%)	-	
Bupivacaine (epidural)	26/52 (50%)	-	
Bupivacaine 0.5% +	-	40/80 (50%)	
fentanyl			
Bupivacaine 0.5% +	-	40/80 (50%)	
morphine			
Vasopressor strategy			
Phenylephrine infusion	-	40/80 (50%)	
Ephedrine boluses	-	40/80 (50%)	

Values are mean ± SD or *n* (%).

**Table 2:** Hemodynamic parameters in patients receiving epidural analgesia (EA) or spinal anesthesia (SA).

Parameter	EA (*n* = 52)	SA (*n* = 80)	*p*
CI (L/min/m^2^)	3.9 ± 0.6	3.9 ± 0.6	0.530
SV (mL)	85.1 ± 11.3	78.1 ± 9.7	<0.001
HR (bpm)	85.8 ± 12.5	92.2 ± 12.9	0.009
MAP (mmHg)	95.7 ± 8.6	92.8 ± 9.3	0.070
SVRI (dyn·s·cm^−5^/m^2^)	1988 ± 343	2014 ± 418	0.714
TFC (1/kΩ)	21.7 ± 4.4	21.8 ± 3.2	0.881

**Table 3:** Spinal anesthesia (SA)—hemodynamic parameters by intrathecal opioid additive.

Parameter	Bupivacaine +	Bupivacaine +	*p*
	Fentanyl (*n* = 40)	Morphine (*n* = 40)	
CI (L/min/m^2^)	4.0 ± 0.7	3.7 ± 0.6	0.063
SV (mL)	78.0 ± 9.6	78.5 ± 10.1	0.827
HR (bpm)	94.6 ± 12.8	88.9 ± 13.5	0.055
MAP (mmHg)	92.6 ± 10.4	92.9 ± 8.2	0.879
SVRI (dyn·s·cm^−5^/m^2^)	1930.8 ± 432.5	2093.1 ± 392.7	0.087
TFC (1/kΩ)	21.6 ± 3.0	22.0 ± 3.5	0.599

Values are mean ± SD.

**Table 4:** Spinal Anesthesia (SA)—hemodynamic parameters by vasopressor strategy.

Parameter	Phenylephrine (*n* = 40)	Ephedrine (*n* = 40)	*p*
CI (L/min/m^2^)	3.7 ± 0.6	4.0 ± 0.6	0.017
SV (mL)	77.7 ± 9.7	78.6 ± 9.9	0.703
HR (bpm)	89.2 ± 11.9	95.3 ± 13.4	0.031
MAP (mmHg)	95.4 ± 8.4	90.8 ± 9.3	0.024
SVRI (dyn·s·cm^−5^/m^2^)	2153.4 ± 364.9	1886.3 ± 399.8	0.003
TFC (1/kΩ)	21.9 ± 3.9	21.8 ± 2.6	0.972

**Table 5:** Multivariable logistic regression for hypotension (≥20% SBP drop).

Covariate	Adjusted OR (OR)	95% CI (OR)	Effect (β)	S.E.	*p*	R^2^
Model (global)					0.013	0.124
SA vs. EA	3.48	1.55–7.83	1.248	0.413	0.003	
MAP (per 1 mmHg)	1.05	1.00–1.09	0.047	0.022	0.032	
CI (per 1 L·min^−1^·m^−2^)	1.23	0.66–2.31	0.208	0.321	0.517	
HR (per 1 bpm)	1.00	0.96–1.03	−0.005	0.016	0.756	

## Data Availability

The data presented in this study are available on request from the corresponding author.

## Abbreviations

The following abbreviations are used in this manuscript:

BP	Blood pressure
CI	Cardiac Index
CO	Cardiac Output
DBP	Diastolic blood pressure
EA	Epidural analgesia
HR	Heart rate
ICG	Impedance cardiography
MAP	Mean arterial pressure
OR	Odds ratios
SA	Spinal anesthesia
SBP	Systolic blood pressure
SQI	Signal Quality Index
SV	Stroke volume
SVRI	Systemic vascular resistance index
TFC	Thoracic fluid content
